# Ozone Exposure During Pregnancy and Risk of Gestational Hypertension or Preeclampsia in China

**DOI:** 10.1001/jamanetworkopen.2023.6347

**Published:** 2023-04-03

**Authors:** Yukai Cheng, Pengpeng Wang, Liyi Zhang, Huijing Shi, Jiufeng Li, Xia Meng, Xirong Xiao, Haixia Dai, Yunhui Zhang

**Affiliations:** 1 Key Laboratory of Health Technology Assessment, National Health Commission of the People’s Republic of China, Fudan University, Shanghai, China; 2 Key Laboratory of Public Health Safety, Ministry of Education, School of Public Health, Fudan University, Shanghai, China; 3 Department of Obstetrics and Gynecology, Obstetrics and Gynecology Hospital of Fudan University, Shanghai, China; 4 State Environmental Protection Key Laboratory of Formation and Prevention of Urban Air Pollution Complex, Shanghai Academy of Environmental Sciences, Shanghai, China

## Abstract

**Importance:**

Although certain air pollutants have been associated with adverse obstetric outcomes, evidence regarding the association of ozone (O_3_) exposure with the risk of hypertensive disorders in pregnancy (HDP) is limited and inconsistent.

**Objectives:**

To evaluate the association between gestational O_3_ exposure and HDP (ie, gestational hypertension and preeclampsia) risk, and to explore the window of susceptibility for O_3_ exposure during pregnancy.

**Design, Setting, and Participants:**

This cohort study recruited pregnant patients from the Obstetrics and Gynecology Hospital of Fudan University in Shanghai, China, from March 2017 to December 2018. Participants were older than 18 years, had no infectious diseases or chronic noncommunicable diseases before pregnancy, were Shanghai residents with intent to participate in the study, and had plans to give birth in Shanghai. Gestational hypertension and preeclampsia were diagnosed according to the diagnostic criteria of the Chinese Society of Obstetrics and Gynecology during the study period. Data on residential addresses, demographic characteristics, and household living environments were collected from participants through a questionnaire survey. Data were analyzed from December 10, 2021, to May 10, 2022.

**Exposures:**

A high temporospatial resolution model was applied to predict individual levels of daily O_3_ exposure during pregnancy.

**Main Outcomes and Measures:**

The outcomes were gestational hypertension and preeclampsia, and data on these diagnoses were extracted from the hospital’s information system. A logistic regression model was used to estimate the associations between O_3_ exposure and risk of gestational hypertension or preeclampsia. Exposure-response associations were confirmed by restricted cubic spline functions. Distributed lag models were used to identify the O_3_ exposure window of susceptibility.

**Results:**

Among the 7841 participants (all females; mean [SD] age, 30.4 [3.8] years), 255 (3.2%) had gestational hypertension and 406 (5.2%) had preeclampsia. Pregnant individuals with HDP had considerably higher prepregnancy body mass indexes and lower educational levels. The mean (SD) O_3_ exposure levels were 97.66 (25.71) μg/m^3^ in the first trimester and 106.13 (22.13) μg/m^3^ in the second trimester. Each 10-μg/m^3^ increment of O_3_ exposure during the first trimester was associated with higher gestational hypertension risk (relative risk, 1.28; 95% CI, 1.04-1.57). However, gestational O_3_ exposure was not associated with the risk of preeclampsia. The restricted cubic spline function analysis revealed an exposure-response association between O_3_ exposure and risk of gestational hypertension.

**Conclusions and Relevance:**

Results of this study showed an association between increased gestational hypertension risk and O_3_ exposure during the first trimester. Furthermore, gestational weeks 1 to 9 were identified as the window of susceptibility for O_3_ exposure and elevated gestational hypertension risk. Sustainable O_3_ control is needed to reduce the disease burden of gestational hypertension.

## Key Points

**Question:**

Is ozone (O_3_) exposure during pregnancy associated with higher risks of hypertensive disorders in pregnancy?

**Findings:**

In this cohort study in China involving 7841 pregnant individuals, O_3_ exposure in the first trimester was associated with increased gestational hypertension risk but was not associated with preeclampsia risk. Gestational weeks 1 to 9 were identified as the window of susceptibility for O_3_ exposure and elevated gestational hypertension risk.

**Meaning:**

Findings of this study suggest the need for better and sustainable O_3_ control to reduce the disease burden of gestational hypertension among pregnant individuals.

## Teaser

This cohort study examines the association of exposure to ozone with gestational hypertension and preeclampsia among pregnant individuals in Shanghai, China.

## Introduction

Hypertensive disorders in pregnancy (HDP) are a group of gestational complications that include 4 clinical phenotypes: chronic hypertension, gestational hypertension, preeclampsia-eclampsia, and chronic hypertension superimposed on preeclampsia.^1^ Worldwide, the prevalence of HDP ranges from 5.2% to 8.2%.^2^ In China, the prevalence of HDP varies by region, ranging from 5% to 10%.^3^ Hypertensive disorders in pregnancy have been shown to exert substantial adverse effects on both maternal and fetal health. Individuals with HDP experience higher risks of subclinical atherosclerosis, chronic hypertension, diabetes, dyslipidemia, kidney dysfunction, and other cardiovascular conditions.^4^ For example, individuals with HDP had a 2-fold risk of cardiovascular disease readmission within 3 years of delivery and up to a 10-fold risk of high blood pressure within 10 years of delivery.^5^ In addition, fetal growth restriction and increased cardiovascular risk in infants may also be associated with maternal HDP.^6,7^

Several risk factors for HDP, including obesity, multiple pregnancies, anemia, maternal age, and family history of hypertension, have been identified in past investigations.^2,8^ An increasing number of studies have revealed that exposure to air pollutants, such as fine particulate matter (PM_2.5_) and nitrogen dioxide (NO_2_), was associated with a higher risk of hypertension in the general population.^9,10^ Pregnancy is a unique period for individuals, producing rapid changes in blood volume, cardiac output, and maternal heart rate.^11^ Compared with the general population, pregnant individuals are more vulnerable to environmental contaminants. In addition to PM_2.5_ and NO_2_ pollution, ozone (O_3_) pollution has become a major threat to human health, with higher O_3_ levels brought on by chronic climate change and increasing anthropogenic O_3_ precursor emissions.^12^ As the primary pollutant in the Yangtze River Delta region of China in 2017, the proportion of O_3_ reached 50.4% for the first time, surpassing that of PM_2.5_ (44.5%); of the primary pollutants, the proportion of O_3_ ranked first from 2017 to 2022.^13^

Two similar meta-analyses reviewed the association of ambient air pollution with HDP risk and derived inconsistent conclusions about the implications of O_3_ exposure.^14,15^ Specifically, Hu et al^14^ found that increases in the risk of HDP were associated with exposure to O_3_ during the first trimester. However, Pedersen et al^15^ showed that the association of O_3_ exposure with an increased risk of combined pregnancy-induced hypertensive disorders and preeclampsia was not significant. A growing body of literature has found an association between O_3_ exposure during pregnancy and HDP risk.^16,17,18^ For instance, a cohort study of 655 529 participants in Florida showed that exposure to O_3_ could play a role in increased HDP risk, and early pregnancy appeared to be a potential window of susceptibility for exposure.^16^ A retrospective case-control study in the US and a registry-based study in Japan showed similar associations between maternal O_3_ exposure and HDP risk.^17,18^ In contrast, some studies from South Korea and China did not show an association of O_3_ exposure with HDP risk.^19,20^ This inconsistency may be due to different populations, different exposure assessment approaches, unadjusted residual confounders, selection bias, or the levels of O_3_ pollution across study areas.

Instead of investigating gestational hypertension and preeclampsia separately, most environmental health studies combined these conditions as HDP, ignoring the differences between diseases. Thus, based on a prospective cohort in Shanghai, China, in this cohort study, we aimed to evaluate the association between gestational O_3_ exposure and HDP (ie, gestational hypertension and preeclampsia) risk and to explore the window of susceptibility for O_3_ exposure during pregnancy.

## Methods

### Study Population

This population-based cohort study was based on medical information from the Obstetrics and Gynecology Hospital of Fudan University, for which the catchment area is the city of Shanghai. The ethics committee of the Obstetrics and Gynecology Hospital of Fudan University approved the study protocol. Written informed consent was obtained from all participants. We followed the Strengthening the Reporting of Observational Studies in Epidemiology (STROBE) reporting guideline.

Pregnant individuals, who subsequently had singleton births at the Obstetrics and Gynecology Hospital of Fudan University, were recruited between March 2017 and December 2018.^21^ The eligibility criteria included age older than 18 years, no infectious diseases or chronic noncommunicable diseases before pregnancy, Shanghai residency with intent to participate in the study, and plan to give birth in Shanghai. All participants were of Han Chinese ethnicity. Participants were asked to complete a questionnaire, which collected data on their residential address, demographic characteristics, and household living environment during routine pregnancy checkups at the hospital in early pregnancy (10-14 weeks of gestation). The questionnaire was distributed by medical staff who were trained in standardized practices and who informed the participants of study requirements and instructions.

The study flowchart is shown in eFigure 1 in Supplement 1. Participants’ residential addresses covered the entire Shanghai municipal city, showing a radial distribution with the city center as the focus (eFigure 2 in Supplement 1).

### Outcomes, Exposures, and Covariates

The outcomes were gestational hypertension and preeclampsia, which were diagnosed according to the diagnostic criteria of the Chinese Society of Obstetrics and Gynecology during the study period.^22^ Data on gestational hypertension and preeclampsia diagnostic test results were extracted from the hospital’s information system. Gestational hypertension was defined as elevated blood pressure without evidence of proteinuria after 20 weeks of pregnancy. Preeclampsia was defined as the onset of hypertension along with proteinuria after 20 weeks of gestation.^23^

Individual levels of daily 8-hour maximum moving-average O_3_ exposure with a 1 km × 1 km resolution were predicted using a full–temporospatial coverage model, based on residential address, developed by Meng et al.^24^ This machine learning model uses the random forest algorithm to incorporate predictors, including O_3_ simulations from the Community Multiscale Air Quality chemical transport, meteorological parameters, population density, road network data, and elevation data. The individual daily mean PM_2.5_ exposure was assessed by a random forest model, which was also developed by Meng et al.^25^ The relative humidity, temperature, and data on 4 other environmental air pollutants (carbon monoxide [CO], NO_2_, particulate matter with diameter ≤10 μm [PM_10_], and sulfur dioxide [SO_2_]) with a 15 km ×15 km resolution were obtained from the Science Data Bank.^26^ Given that HDP generally begins to appear after week 20 of pregnancy, to ensure the accuracy of exposure time, we calculated the individual mean concentrations of air pollutants in the following 2 periods: first trimester (weeks 1-12) and second trimester (weeks 13-27).

Covariates were determined a priori based on previous studies, including maternal age (<26, 26-34, and ≥35 years), prepregnancy body mass index (BMI [calculated as weight in kilograms divided by height in meters squared]; <18.5, 18.5-23, and ≥24), maternal educational level (<13, 13-16, and ≥17 years), season of conception (spring, summer, autumn, or winter), alcohol consumption (yes or no), passive smoking (yes or no), and parity (nulliparous or multiparous).^16,27^ Studies have shown that temperature and humidity are associated with gestational hypertension, preeclampsia, and blood pressure fluctuations.^28,29,30^ Therefore, temperature and relative humidity were included in the model as confounders.

### Statistical Analysis

Descriptive analyses were performed to summarize the demographic characteristics of participants, exposure levels of air pollutants, and meteorological data in this study. Pearson correlation was used to estimate the correlations among O_3_, PM_2.5_, CO, NO_2_, PM_10_, and SO_2_ exposures during each trimester.

Logistic regression models were used to estimate the association between O_3_ exposure in each trimester and risk of gestational hypertension or preeclampsia, and the estimated relative risk (RR) was interpreted as the disease risk for each 10-μg/m^3^ increment in O_3_ exposure. Restricted cubic spline function–based logistic regression models were used to visualize the concentration-response curve between O_3_ exposure and gestational hypertension or preeclampsia risk. To identify the window of susceptibility for exposure, distributed lag models were applied to estimate the associations between HDP (gestational hypertension and preeclampsia) risk and weekly O_3_ exposures, reducing the role of collinearity in O_3_ exposures during other weeks by creating a flexible cross-basis function.^31^ Natural cubic spline functions with 3 degrees of freedom were assigned to the exposure variable in the value and lag dimensions.

Additionally, there is evidence that other air pollutants are associated with HDP risk.^32,33^ Two-pollutant models were used to examine potential confounding from copollutants. The 2-pollutant models included 1 of 5 other air pollutants as a confounder to adjust the previous model to verify the robustness of the association between O_3_ exposure and HDP risk. We further performed stratified analyses for the week-specific association by maternal age and prepregnancy BMI.

All analyses were performed using R, version 4.20 (R Foundation for Statistical Computing). *P* < .05 was considered to be statistically significant, and all statistical tests were 2-sided. Data were analyzed from December 10, 2021, to May 10, 2022.

## Results

Of the 8718 pregnant individuals recruited, 7841 (all females; mean [SD] age, 30.4 [3.8] years) were included in the current analysis. Table 1 demonstrates the general characteristics of these participants. A total of 255 participants (3.2%) were diagnosed with gestational hypertension and 406 (5.2%) with preeclampsia. Compared with pregnant individuals without HDP (7180 [91.6%]), those with HDP had significantly higher prepregnancy BMIs and lower educational levels. Participants who conceived during spring and were nulliparous were more likely to be diagnosed with gestational hypertension or preeclampsia. No significant differences were found in maternal age, alcohol consumption, or passive smoking among the 3 groups (gestational hypertension, preeclampsia, and without HDP).

**Table 1.** Characteristics of Study Participants (N = 7841)

Characteristic	No. (%)			χ^2^	*P* value
	Gestational hypertension (n = 255)	Preeclampsia (n = 406)	Without HDP (n = 7180)		
Maternal age, y					
<26	16 (6.3)	31 (7.6)	531 (7.4)	6.00	.20
26-34	200 (78.4)	301 (74.1)	5634 (78.5)		
≥35	39 (15.3)	74 (18.2)	1015 (14.1)		
Prepregnancy BMI					
<18.5	13 (5.1)	28 (6.9)	1119 (15.6)	198.30	<.001
18.5-23	157 (61.6)	242 (59.6)	5075 (70.7)		
≥24	85 (33.3)	136 (33.5)	986 (13.7)		
Maternal educational level, y					
<13	12 (4.7)	34 (8.4)	511 (7.1)	16.69	.002
13-16	207 (81.9)	314 (77.3)	5231 (72.9)		
≥17	36 (14.1)	58 (14.3)	1438 (20.0)		
Season of conception					
Spring	72 (28.2)	105 (25.8)	1533 (21.4)	34.17	<.001
Summer	29 (11.4)	99 (24.4)	1309 (18.2)		
Autumn	52 (20.4)	88 (21.7)	1734 (24.2)		
Winter	102 (40.0)	114 (28.1)	2604 (36.3)		
Alcohol consumption					
No	240 (94.1)	371 (91.4)	6533 (91.0)	3.01	.22
Yes	15 (5.9)	35 (8.6)	647 (9.0)		
Passive smoking					
No	231 (90.6)	346 (85.2)	6320 (88.0)	4.56	.10
Yes	24 (9.4)	60 (14.8)	860 (12.0)		
Parity					
Nulliparous	218 (85.5)	351 (86.5)	5442 (75.8)	35.89	<.001
Multiparous	37 (14.5)	55 (13.6)	1738 (24.2)		

Abbreviations: BMI, body mass index (calculated as weight in kilograms divided by height in meters squared); HDP, hypertensive disorders in pregnancy.

### Distribution of O_3_ and Other Air Pollutants

Table 2 describes the distribution of air pollutant exposure and meteorological factors during the first 2 trimesters. The mean (SD) concentrations of pollutants during pregnancy ranged from 97.66 (25.71) to 106.13 (22.13) μg/m^3^ for O_3_, 37.93 (7.16) to 41.18 (8.49) μg/m^3^ for PM_2.5_, 0.73 (0.13) to 0.68 (0.12) mg/m^3^ for CO, 40.95 (9.22) to 43.67 (9.88) μg/m^3^ for NO_2_, 57.88 (10.28) to 60.34 (10.50) μg/m^3^ for PM_10_, and 11.83 (2.19) to 12.70 (2.59) μg/m^3^ for SO_2_. Except for O_3_, other air pollutants had a higher exposure concentration in the first trimester than in the second trimester. Inverse correlations were found between gestational exposure to O_3_ and other air pollutants (Pearson correlation coefficient range, −0.77 to −0.33), and there were correlations among other air pollutants (Pearson correlation coefficient range, 0.52 to 0.86) (eFigure 3 in Supplement 1).

**Table 2.** Distribution of Air Pollutant Concentrations, Temperature, and Relative Humidity During Pregnancy

Gestational period	Mean (SD) [range]	Median (IQR)
**First trimester**		
Air pollutants		
O_3_, μg/m^3^	97.66 (25.71) [55.26-143.81]	96.07 (72.98-123.31)
PM_2.5_, μg/m^3^	41.18 (8.49) [23.04-64.08]	42.16 (34.00-48.36)
CO, mg/m^3^	0.73 (0.13) [0.27-1.24]	0.71 (0.63-0.81
NO_2_, μg/m^3^	43.67 (9.88) [7.02-71.71]	44.25 (34.89-50.50)
PM_10_, μg/m^3^	60.34 (10.50) [23.09-99.71]	61.64 (52.39-66.83)
SO_2_, μg/m^3^	12.70 (2.59) [4.08-21.23]	12.47 (10.81-14.62)
Temperature, °C	15.85 (7.74) [5.75-30.09]	13.66 (8.62-23.24)
Relative humidity, %	73.85 (3.31) [64.3-81.36]	73.70 (71.36-76.69)
**Second trimester**		
Air pollutants		
O_3_, μg/m^3^	106.13 (22.13) [60.48-143.49]	112.88 (87.07-123.12)
PM_2.5_, μg/m^3^	37.93 (7.16) [22.49-63.65]	37.12 (32.09-43.66)
CO, mg/m^3^	0.68 (0.12) [0.20-1.20]	0.66 (0.60-0.75)
NO_2_, μg/m^3^	40.95 (9.22) [7.26-70.33]	40.02 (33.33-47.16)
PM_10_, μg/m^3^	57.88 (10.28) [27.45-95.70]	58.10 (49.16-65.28)
SO_2_, μg/m^3^	11.83 (2.19) [3.32-18.29]	11.58 (10.17-13.15)
Temperature, °C	18.63 (6.80) [5.37-33.99]	19.31 (12.67-24.88)
Relative humidity, %	74.63 (3.17) [59.67-87.07]	75.15 (72.02-77.41)

Abbreviations: CO, carbon monoxide; NO_2_, nitrogen dioxide; O_3_, ozone; PM_2.5_, fine particulate matter ≤2.5 μm; PM_10_, particulate matter with diameter ≤10 μm; SO_2_, sulfur dioxide.

### Associations Between O_3_ Exposure and HDP Risk

**Figure 1.**
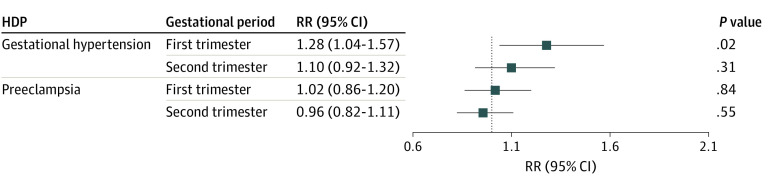
Associations of Ozone Exposure With Risk of Gestational Hypertension or Preeclampsia Exposure was the relative risk (RR) for each 10-μg/m^3^ increase. Models were adjusted for maternal age, prepregnancy body mass index, maternal educational level, season of conception, alcohol consumption, passive smoking, parity, temperature, and relative humidity. HDP indicates hypertensive disorders in pregnancy.

Figure 1 shows the association of HDP risk with O_3_ exposure during pregnancy by multivariate logistic regression. Gestational hypertension risk was associated with O_3_ exposure in the first trimester for each 10-μg/m^3^ increase in O_3_ (RR, 1.28; 95% CI, 1.04-1.57). No association between O_3_ exposure in each trimester and preeclampsia risk was found.

**Figure 2.**
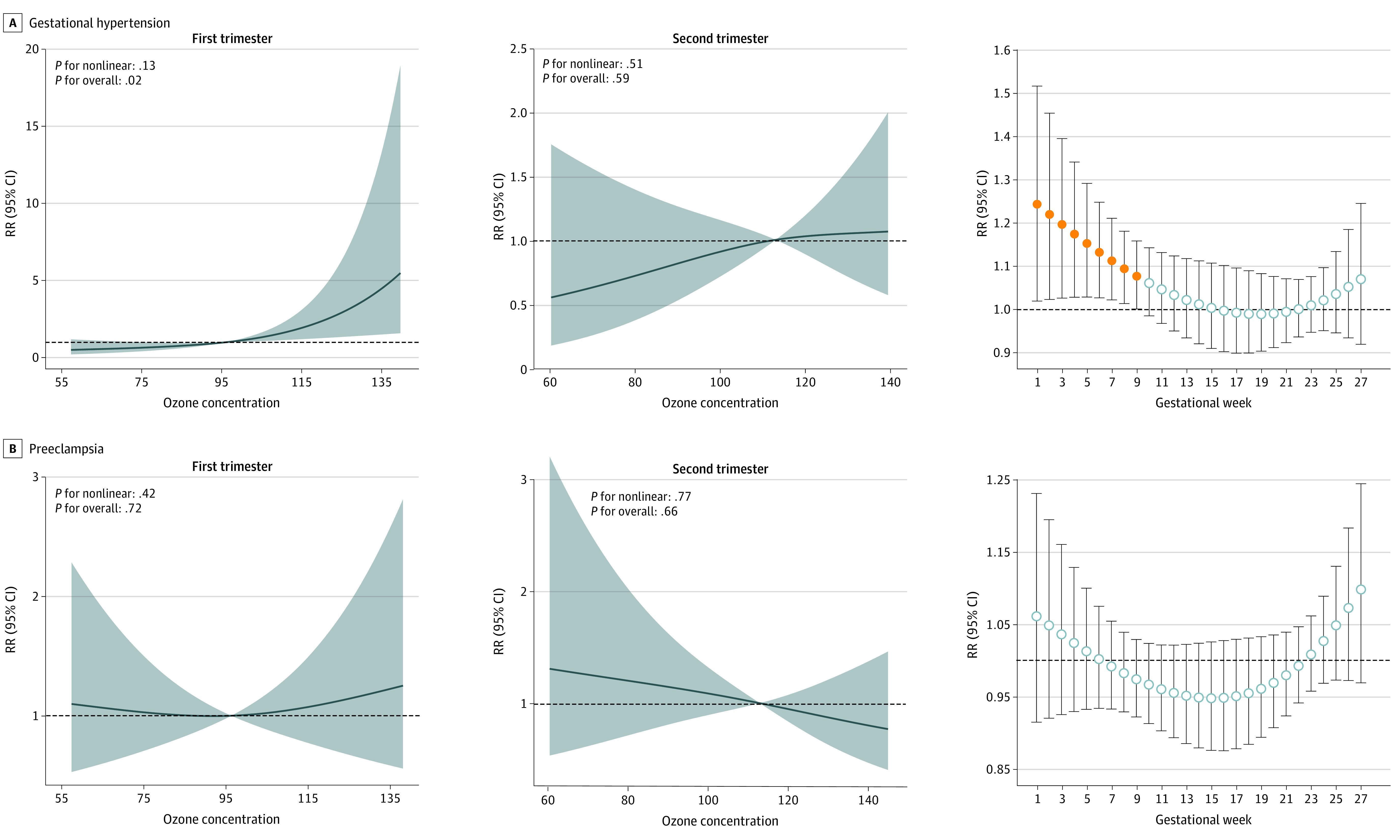
Exposure-Response Associations Between Ozone Exposure and Gestational Hypertension or Preeclampsia and Relative Risk (RR) for Gestational Hypertension or Preeclampsia With Weekly Ozone Exposure Models were adjusted for maternal age, prepregnancy body mass index, maternal educational level, season of conception, alcohol consumption, passive smoking, parity, temperature, and relative humidity. Gray-shaded ribbon indicates 95% CIs. Dashed lines, relative risk = 1; solid circles, window of susceptibility for ozone exposure; open circles, no significant associations between gestational weekly ozone exposure and risk of gestation hypertension or preeclampsia.

Figure 2 presents the exposure-response associations between O_3_ exposure and HDP risk. The risk of gestational hypertension was associated with increasing O_3_ concentration during the first trimester. No exposure-response association between O_3_ exposure and preeclampsia risk was observed. Results of the distributed lag models showed associations between gestational hypertension and O_3_ exposure during gestational weeks 1 to 9. No associations were observed between gestational weekly O_3_ exposure and preeclampsia risk.

### Sensitivity Analyses

**Figure 3.**
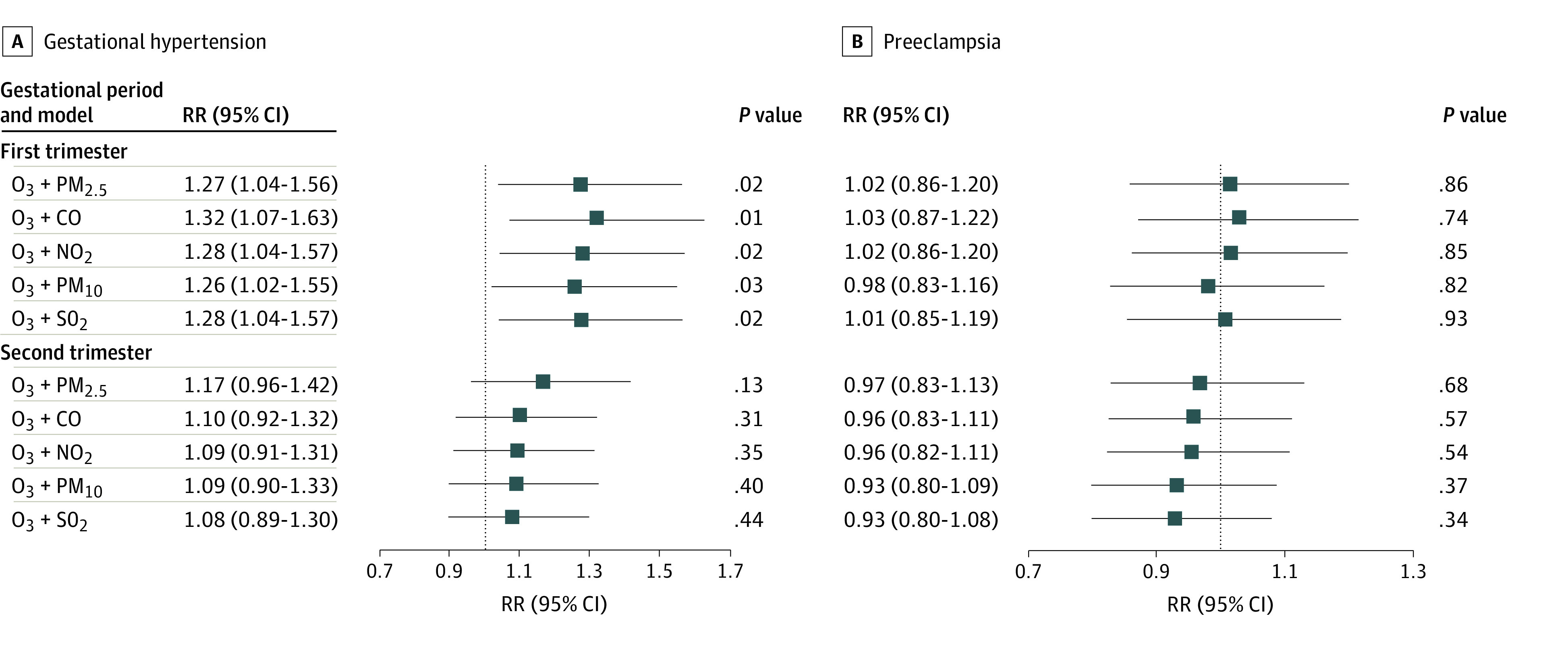
Associations of Ozone (O_3_) Exposure With Risk of Gestational Hypertension or Preeclampsia in 2-Pollutant Models Exposure was the relative risk (RR) for each 10-μg/m^3^ increase. Models were adjusted for maternal age, prepregnancy body mass index, maternal educational level, season of conception, alcohol consumption, passive smoking, parity, temperature, and relative humidity. CO indicates carbon monoxide; NO_2_, nitrogen dioxide; PM_2.5_, fine particulate matter with a diameter of 2.5 μm or smaller; PM_10_, particulate matter with diameter of 10 μm or smaller; RR, relative risk; and SO_2_, sulfur dioxide.

In the 2-pollutant models, the results showed that associations between O_3_ exposure in the first trimester and gestational hypertension risk were robust after adjusting for PM_2.5_, CO, NO_2_, PM_10_, and SO_2_ (Figure 3). After correcting for other air pollutants, we still found no associations between O_3_ exposure in different trimesters and preeclampsia risk.

When stratified by maternal age, we observed that O_3_ exposure was associated with elevated gestational hypertension risk in participants aged 26 to 34 years, and the identified window of susceptibility for exposure was gestational weeks 21 to 23. In addition, O_3_ exposure was associated with increased preeclampsia risk among participants 35 years or older. In the prepregnancy BMI stratified analysis, associations between O_3_ exposure and gestational hypertension risk were found among individuals with a prepregnancy BMI ranging from 18.5 to 23. No association was observed between O_3_ exposure and preeclampsia risk at different prepregnancy BMIs (eFigure 4 in Supplement 1).

## Discussion

As a megacity in the Yangtze River Delta region, Shanghai has O_3_ pollution in the warm season (spring through summer), and the O_3_ concentration in the warm season has been higher than the World Health Organization guidance value of 60 μg/m^3^ in recent years.^34^ In this study, we used a machine learning approach to estimate individual O_3_ exposure in pregnant patients. The mean concentrations of O_3_ during pregnancy ranged from 97.66 to 106.13 μg/m^3^, which were greater than the levels found in studies from the US, Korea, Japan, and China (Guangzhou and Wuhan).^16,19,27,35,36^ The method we used to assess individual O_3_ exposure outperformed previous exposure assessment methods in terms of exposure accuracy, which may also account for the differences in the results.^18,19,35^

In the present study, we found associations between gestational hypertension risks and O_3_ exposure, but no association was found between O_3_ exposure and preeclampsia risk. The association of O_3_ exposure with the risk of gestational hypertension observed in this study was in accordance with findings in the Wuhan, China, cohort study (n = 38 115; odds ratio, 1.05 [95% CI, 1.02-1.07] per 10 μg/m^3^).^35^ Accordingly, the Korean National Health Insurance Service and National Sample Cohort cohort study (n = 18 565) from South Korea showed no association between prenatal O_3_ exposure and preeclampsia risk.^19^ Nevertheless, a US study from Allegheny County, Pennsylvania, revealed that O_3_ exposure in early pregnancy was not associated with an elevated risk of gestational hypertension or preeclampsia.^37^ A cohort study in Sweden found that O_3_ exposure even during the first trimester was associated with an increased risk of preeclampsia.^38^ These inconsistent results can be explained by the O_3_ pollution levels in different countries and regions. It has been reported that a nontoxic dose of O_3_ can activate the antioxidant system, enhance the immune system response by increasing the production of interferon, and reduce tissue hypoxia.^39^ Therefore, the damage of cardiovascular diseases associated with lower O_3_ exposure may be minimal and difficult to show.^40,41^

Additionally, the identification of windows of susceptibility for O_3_ exposure and elevated HDP risk is of great importance to clinical and public health, not only helping to improve understanding of potential biological mechanisms but also providing information for the implementation of targeted and effective prevention strategies. Few studies have identified windows for O_3_ exposure and HDP risk at the weekly level. A study conducted in Florida showed that O_3_ exposure during pregnancy was associated with an increased risk of HDP, and gestational weeks 1 to 24 may be the potentially crucial window of O_3_ exposure.^16^ In the present study, the windows of susceptibility for O_3_ exposure were narrower than those in the Florida study. Gestational weeks 1 to 9 were identified as the window for O_3_ exposure during pregnancy. Although there are differences in susceptibility windows, those for O_3_ exposure in early pregnancy were all found. In pregnant individuals, the physiological system begins to change in the early stage of pregnancy and may be extremely sensitive to environmental pollution.^42^ Air pollutant exposure may be associated with an altered normal blood pressure pattern and gestational hypertension.^43,44^

Furthermore, the association of O_3_ exposure with elevated gestational hypertension risk presented in participants aged 26 to 34 years, and the association of O_3_ exposure with increased preeclampsia risk was seen in those 35 years or older. Some studies have shown a 30% increased risk of preeclampsia in pregnant individuals 35 years or older,^8^ which may be explained by an increase in susceptibility to the outcomes of air pollutant exposure at an advanced maternal age. Similarly, toxic kinetics may be age dependent. Compared with older mothers, younger mothers may be more capable of detoxifying and excreting toxic compounds.^45^ Therefore, older mothers may be less resilient to the threat of O_3_ exposure, resulting in an increased risk of HDP. It is generally believed that individuals with higher BMI before pregnancy have a higher risk of developing HDP.^46,47^ However, in this study, the association between O_3_ exposure and gestational hypertension risk was observed only in individuals whose prepregnancy BMI ranged from 18.5 to 23. Existing evidence in individuals with healthy prepregnancy BMI indicated that excessive weight gain during pregnancy, which may be associated with changes in air pollution, diet, and physical activity, may play a role in increased HDP risk through oxidative stress.^48,49,50^

It is well known that air pollution poses a substantial cardiovascular risk, and the mechanisms of cardiovascular diseases that are associated with air pollution include inflammation, oxidative stress, and endothelial dysfunction.^51^ Given the similarities between HDP and cardiovascular diseases, it is plausible that HDP may share common pathogenic pathways with cardiovascular diseases that are induced by air pollutants.^35,52^ Animal experiments showed that the developing placenta was an indirect target of inhaled O_3_ and that systemic maternal cardiovascular abnormalities might be induced by O_3_ exposure during a specific window of gestation.^53^ Pregnant Sprague-Dawley rats exposed to 0.3 ppm of O_3_ at gestational day 10 exhibited several late-stage cardiovascular outcomes that were not evident in gestational day 20–exposed dams, including an elevated uterine artery resistance index and reduced cardiac output and stroke volume.^53^ One epidemiological study has shown that higher concentrations of O_3_ exposure can be a factor in acute vasoconstriction in the general population.^54^ However, due to the physiological changes in pregnant individuals^55^ that make them sensitive to air pollution, the mechanism by which O_3_ affects blood pressure during pregnancy may be more complex than that in the general population, which requires further exploration.

### Limitations

Some limitations should be considered when interpreting the findings of this study. First, although the high-precision and high-resolution model was applied to individual O_3_ exposure assessment, only outdoor exposure assessments were conducted based on residential address, and indoor O_3_ pollution was not considered.^56^ Second, the diagnosis dates of HDP and severe features of preeclampsia were not available for analysis. Future studies with data on the severity of preeclampsia and date of diagnosis are needed to elucidate the association of O_3_ with subgroups of preeclampsia by using more accurate exposure windows.^19^ Third, in addition to the many confounders we considered, there are still some uncontrolled factors. Most participants worked during early pregnancy, and the workplace environment and job type may be potential confounders. Environmental noise and psychosocial pressure also play important roles in blood pressure disorders.^57,58^

## Conclusions

In this hospital-based prospective cohort study, there was an association between O_3_ exposure in the first trimester and increased risk of gestational hypertension. The findings indicated that gestational weeks 1 to 9 were the window of susceptibility for O_3_ exposure and elevated gestational hypertension risk. Sustainable O_3_ control is needed to reduce the disease burden of gestational hypertension.

## Notes

Supplement 1. **eFigure 1.** The Flowchart for Participant Inclusion and Exclusion **eFigure 2.** Spatial Distribution on Residential Address of Study Participants **eFigure 3.** Correlations Between Air Pollutants During Pregnancy **eFigure 4.** O_3_ Exposure (per 10 μg/m^3^) Increase RRs of Gestational Hypertension or Preeclampsia (Relative Risks, 95% CIs) During 1-27 Gestational Weeks Stratified by Maternal Age and Prepregnancy BMI

Supplement 2.
Data Sharing Statement
