# Supplementary material for: Ozone Exposure During Pregnancy and Risk of Gestational Hypertension or Preeclampsia in China
Source: JAMA Netw Open. 2023 Apr 3;6(4):e236347. doi: 10.1001/jamanetworkopen.2023.6347 (PMC10071346; doi:10.1001/jamanetworkopen.2023.6347)
Supplement: Supplement 1. — eFigure 1. The Flowchart for Participant Inclusion and Exclusion eFigure 2. Spatial Distribution on Residential Address of Study Participants eFigure 3. Correlations Between Air Pollutants During Pregnancy eFigure 4. O3 Exposure (per 10 μg/m3) Increase RRs of Gestational Hypertension or Preeclampsia (Relative Risks, 95% CIs) During 1-27 Gestational Weeks Stratified by Maternal Age and Prepregnancy BMI [file jamanetwopen-e236347-s001.pdf]

Supplemental Online Content

Cheng Y, Wang P, Zhang L, et al. Ozone exposure during pregnancy and risk of gestational hypertension or preeclampsia in China. *JAMA Netw Open*. 2023;6(4):e236347. doi:10.1001/jamanetworkopen.2023.6347

eFigure 1. The Flowchart for Participant Inclusion and Exclusion

eFigure 2. Spatial Distribution on Residential Address of Study Participants

eFigure 3. Correlations Between Air Pollutants During Pregnancy

eFigure 4. O_3 Exposure (per 10 μg/m^3) Increase RRs of Gestational Hypertension or Preeclampsia (Relative Risks, 95% CIs) During 1-27 Gestational Weeks Stratified by Maternal Age and Prepregnancy BMI

This supplemental material has been provided by the authors to give readers additional information about their work.

© 2023 Cheng Y et al. *JAMA Network Open.*

eFigure 1. The flowchart for participant inclusion and exclusion

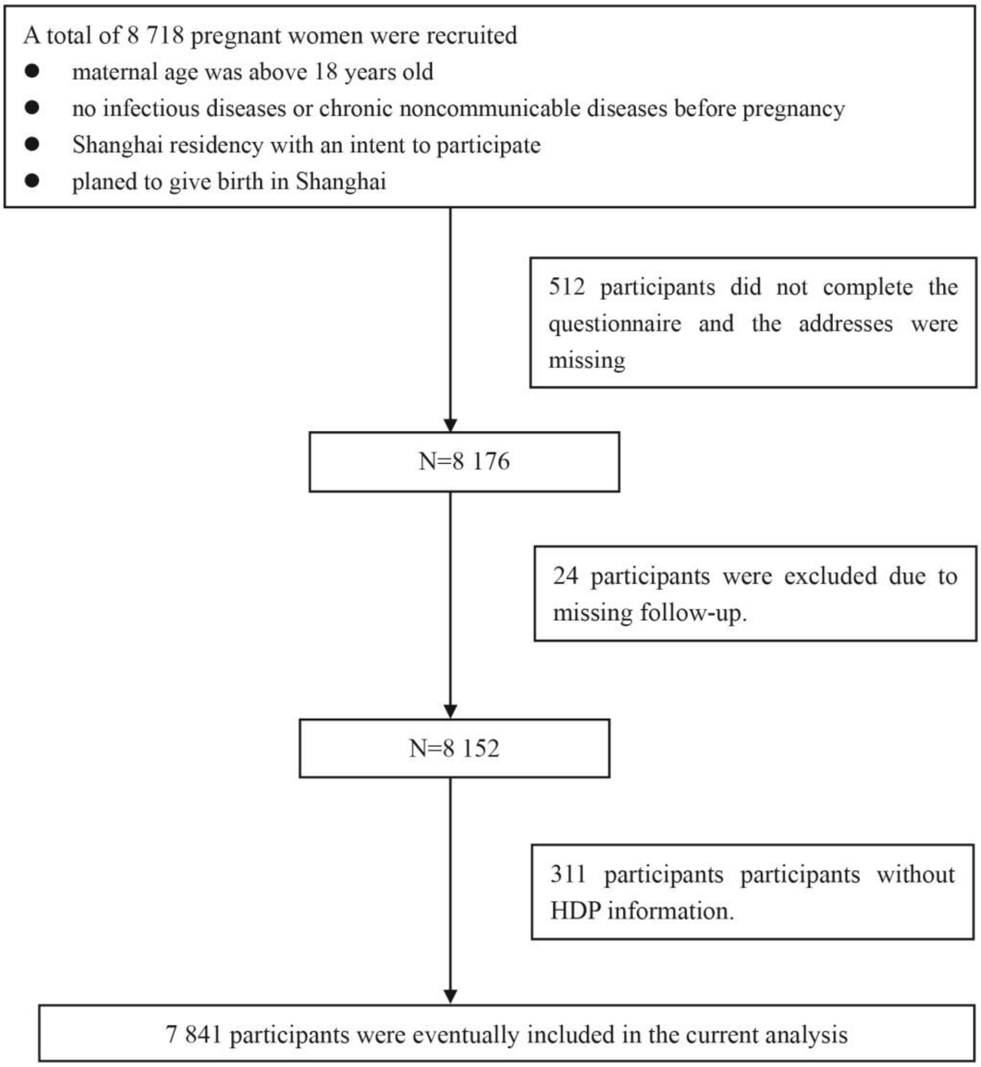

Notes: HDP, hypertensive disorders of pregnancy.

© 2023 Cheng Y et al. *JAMA Network Open.*

eFigure 2. Spatial distribution on residential addresses of the subjects

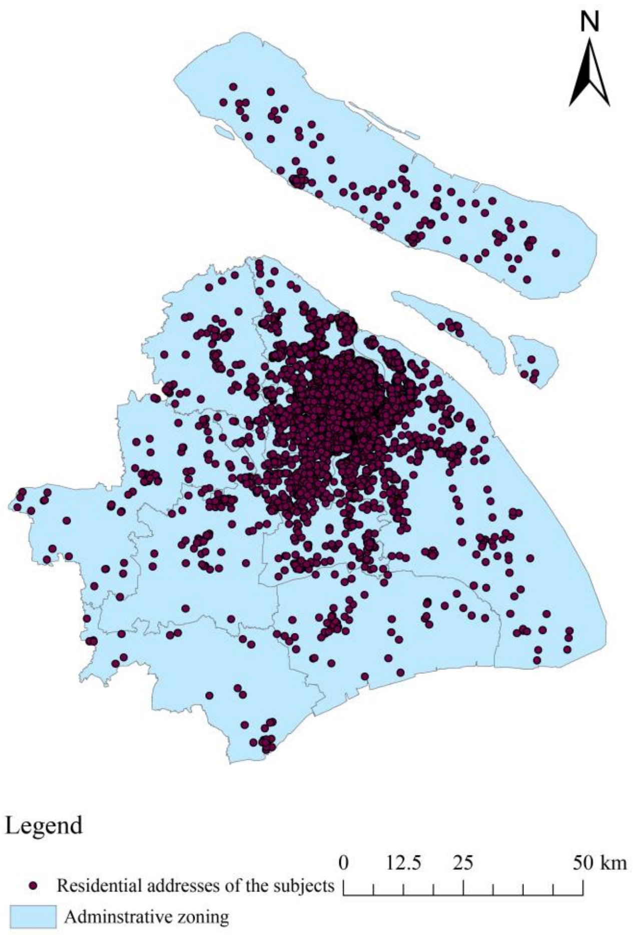

© 2023 Cheng Y et al. *JAMA Network Open.*

eFigure 3. Correlations between air pollutants during pregnancy

O_3	$PM_{2.5}$	CO	NO_2	PM_{10}	SO_2	
1	-0.75	-0.61	-0.77	-0.50	-0.55	O_3
	1	0.63	0.79	0.81	0.65	$PM_{2.5}$
		1	0.85	0.77	0.82	CO
			1	0.86	0.76	NO_2
				1	0.74	PM_{10}
					1	SO_2

First trimester

O_3	$PM_{2.5}$	CO	NO_2	PM_{10}	SO_2	
1	-0.64	-0.50	-0.74	-0.42	-0.34	O_3
	1	0.53	0.79	0.85	0.52	$PM_{2.5}$
		1	0.83	0.74	0.70	CO
			1	0.84	0.72	NO_2
				1	0.75	PM_{10}
					1	SO_2

Second trimester

© 2023 Cheng Y et al. *JAMA Network Open.*

eFigure 4. O_3 exposure (per 10 μg/m^3) increase RRs of gestational hypertension or preeclampsia (Relative Risks, 95% CIs) during 1-27gestational weeks stratified by maternal age and prepregnancy BMI

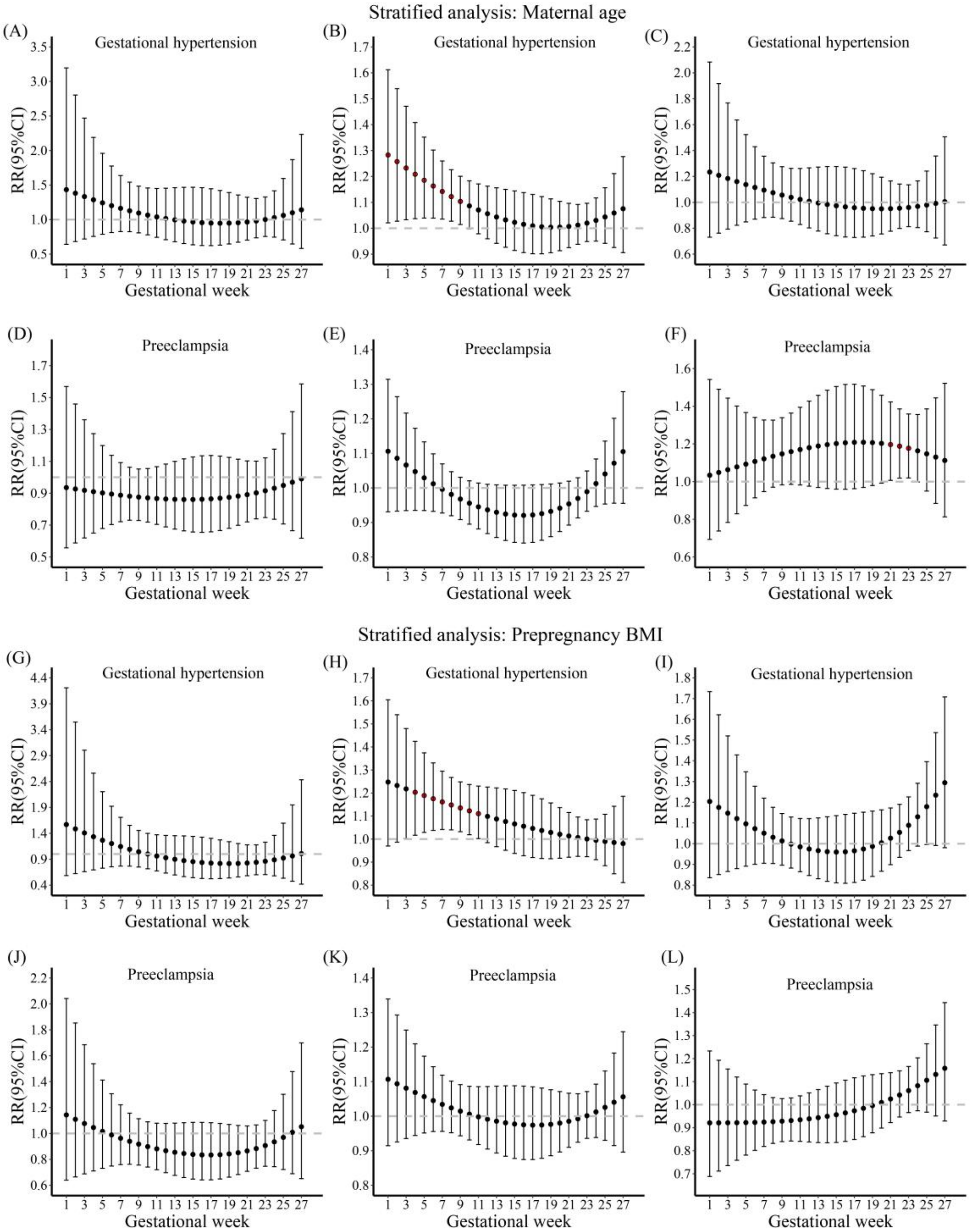

(A), (D) maternal age <26 years; (B), (E) maternal age 26-34 years; (C), (F) maternal age≥35 years; (G), (J) prepregnancy BMI<18.5 kg/m³; (H) (K); prepregnancy BMI 18.5-23 kg/m³; (I), (L) prepregnancy BMI≥24 kg/m³. Models were adjusted for, maternal age, prepregnancy BMI, maternal education level, season of conception, alcohol consumption, passive smoking, parity, temperature and relative humidity.

© 2023 Cheng Y et al. *JAMA Network Open.*
