# Supplementary material for: Ozone Exposure During Pregnancy and Risk of Gestational Hypertension or Preeclampsia in China
Source: JAMA Netw Open. 2023 Apr 3;6(4):e236347. doi: 10.1001/jamanetworkopen.2023.6347 (PMC10071346; doi:10.1001/jamanetworkopen.2023.6347)
Supplement: Supplement 2. — Data Sharing Statement [file jamanetwopen-e236347-s002.pdf]

Data Sharing Statement

Cheng. Ozone Exposure During Pregnancy and Risk of Gestational Hypertension or Preeclampsia in China. *JAMA Netw Open*. Published April 03, 2023. doi:10.1001/jamanetworkopen.2023.6347

Data
Data available: No
